# *N*′-(2,5-Dihydroxy­benzyl­idene)benzene­sulfonohydrazide

**DOI:** 10.1107/S1600536808002225

**Published:** 2008-01-25

**Authors:** Hapipah M. Ali, Juahir Yusnita, Mohd. Razali Rizal, Seik Weng Ng

**Affiliations:** a Department of Chemistry, University of Malaya, 50603 Kuala Lumpur, Malaysia

## Abstract

In the title compound, C_13_H_12_N_2_O_4_S, the dihedral angle between the two aromatic rings is 89.5 (1)°. In the crystal structure, mol­ecules are linked by O—H⋯O_hydr­oxy_ and N—H⋯O_sulfon­yl_ hydrogen bonds, forming a ribbon that propagates along the *b* axis; there is also an intra­molecular O—H⋯N hydrogen bond.

## Related literature

For the structure of 2′-(5-bromo-2-hydroxy­benzyl­idene)­benzene­sulfonohydrazide, see: Ali *et al.* (2007 ▶).
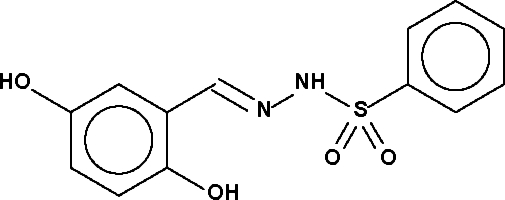


## Experimental

### 

#### Crystal data

C_13_H_12_N_2_O_4_S
*M* *_r_* = 292.31
Monoclinic, 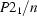

*a* = 12.5814 (2) Å
*b* = 7.1601 (1) Å
*c* = 14.6727 (2) Å
β = 105.540 (1)°
*V* = 1273.46 (3) Å^3^
*Z* = 4
Mo *K*α radiation
μ = 0.27 mm^−1^
*T* = 128 (2) K
0.65 × 0.33 × 0.29 mm

#### Data collection

Bruker APEXII diffractometer
Absorption correction: none
16406 measured reflections
2924 independent reflections
2684 reflections with *I* > 2σ(*I*)
*R*_int_ = 0.023

#### Refinement

*R*[*F* ^2^ > 2σ(*F* ^2^)] = 0.031
*wR*(*F* ^2^) = 0.104
*S* = 1.07
2924 reflections
229 parameters
11 restraints
All H-atom parameters refined
Δρ_max_ = 0.40 e Å^−3^
Δρ_min_ = −0.35 e Å^−3^

### 

Data collection: *APEX2* (Bruker, 2005 ▶); cell refinement: *SAINT* (Bruker, 2005 ▶); data reduction: *SAINT*; program(s) used to solve structure: *SHELXS97* (Sheldrick, 2008 ▶); program(s) used to refine structure: *SHELXL97* (Sheldrick, 2008 ▶); molecular graphics: *X-SEED* (Barbour, 2001 ▶); software used to prepare material for publication: *publCIF* (Westrip, 2008 ▶).

## Supplementary Material

Crystal structure: contains datablocks global, I. DOI: 10.1107/S1600536808002225/wn2236sup1.cif

Structure factors: contains datablocks I. DOI: 10.1107/S1600536808002225/wn2236Isup2.hkl

Additional supplementary materials:  crystallographic information; 3D view; checkCIF report

## Figures and Tables

**Table 1:** Hydrogen-bond geometry (Å, °)

*D*—H⋯*A*	*D*—H	H⋯*A*	*D*⋯*A*	*D*—H⋯*A*
O3—H3O⋯N2	0.85 (1)	1.82 (2)	2.562 (2)	145 (2)
O4—H4O⋯O3^i^	0.85 (1)	1.85 (1)	2.698 (2)	175 (2)
N1—H1N⋯O1^ii^	0.85 (1)	2.05 (1)	2.897 (2)	171 (2)

Symmetry codes: (i) 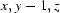; (ii) 
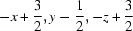
.

**Fig. 1.**
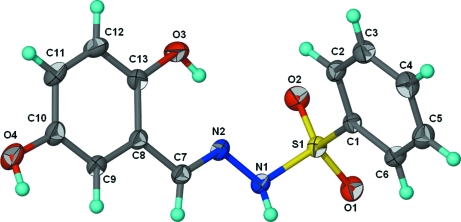
Molecular structure of the title compound. Displacement ellipsoids are drawn at the 70% probability level, and H atoms are shown as spheres of arbitrary radius.

**Fig. 2.**
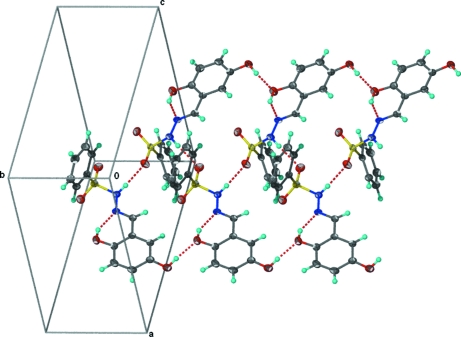
A view of the ribbon structure of the title compound. Dashed lines indicate hydrogen bonds.

## supplementary crystallographic information

### Comment

A recent study reported the crystal structure of 2'-(5-bromo-2-hydroxybenzylidene)benzenesulfonohydrazide (Ali *et al.*, 2007). A second hydroxy group is introduced in the title compound.

### Experimental

Benzenesulfohydrazine (0.29 g, 1.7 mmol) and 2,5-dihydroxybenzaldehyde (0.24 g, 1.7 mmol) were refluxed in ethanol (50 ml) for 2 h. The solvent was removed to give the product Schiff base, and crystals were obtained upon recrystallization from ethanol.

### Refinement

All hydrogen atoms were located in difference Fourier maps. Those bonded to C were restrained to 0.95±0.01 Å, and those bonded to N or O to 0.85±0.01 Å. All displacement parameters were freely refined.

### Crystal data

**Table:**

C_13_H_12_N_2_O_4_S	*F*_000_ = 608
*M**_r_* = 292.31	*D*_x_ = 1.525 Mg m^−^^3^
Monoclinic, *P*2_1_/*n*	Mo *K*α radiation λ = 0.71073 Å
Hall symbol: -P 2yn	Cell parameters from 9918 reflections
*a* = 12.5814 (2) Å	θ = 5.0–63.2º
*b* = 7.1601 (1) Å	µ = 0.27 mm^−^^1^
*c* = 14.6727 (2) Å	*T* = 128 (2) K
β = 105.540 (1)º	Block, yellow
*V* = 1273.46 (3) Å^3^	0.65 × 0.33 × 0.29 mm
*Z* = 4	

### Data collection

**Table:**

Bruker APEXII diffractometer	2684 reflections with *I* > 2σ(*I*)
Radiation source: medium-focus sealed tube	*R*_int_ = 0.023
Monochromator: Graphite	θ_max_ = 27.5º
*T* = 128(2) K	θ_min_ = 1.9º
φ and ω scans	*h* = −16→16
Absorption correction: none	*k* = −9→9
16406 measured reflections	*l* = −18→19
2924 independent reflections	

### Refinement

**Table:**

Refinement on *F*^2^	Secondary atom site location: difference Fourier map
Least-squares matrix: full	Hydrogen site location: inferred from neighbouring sites
*R*[*F*^2^ > 2σ(*F*^2^)] = 0.031	All H-atom parameters refined
*wR*(*F*^2^) = 0.104	*w* = 1/[σ^2^(*F*_o_^2^) + (0.0666*P*)^2^ + 0.482*P*] where *P* = (*F*_o_^2^ + 2*F*_c_^2^)/3
*S* = 1.07	(Δ/σ)_max_ = 0.001
2924 reflections	Δρ_max_ = 0.40 e Å^−^^3^
229 parameters	Δρ_min_ = −0.35 e Å^−^^3^
11 restraints	Extinction correction: none
Primary atom site location: structure-invariant direct methods	

### Special details

**Table:**

Experimental. A medium-focus collimator of 0.8 mm diameter was used on the diffractometer to measure the somewhat large crystal.

### Fractional atomic coordinates and isotropic or equivalent isotropic displacement parameters (Å^2^)

**Table:**

	*x*	*y*	*z*	*U*_iso_*/*U*_eq_	
S1	0.77742 (3)	0.75958 (4)	0.66733 (2)	0.01720 (12)	
N1	0.83576 (9)	0.55528 (16)	0.65868 (8)	0.0190 (2)	
N2	0.84430 (9)	0.52079 (16)	0.56754 (8)	0.0186 (2)	
O1	0.77923 (9)	0.76844 (15)	0.76588 (7)	0.0245 (2)	
O2	0.83351 (8)	0.89724 (14)	0.62689 (7)	0.0249 (2)	
O3	0.85080 (9)	0.64124 (14)	0.40471 (7)	0.0276 (2)	
O4	0.91487 (10)	−0.08605 (15)	0.30407 (8)	0.0307 (3)	
C1	0.63909 (11)	0.74943 (17)	0.59938 (9)	0.0164 (3)	
C2	0.61433 (11)	0.7976 (2)	0.50377 (9)	0.0198 (3)	
C3	0.50524 (12)	0.7887 (2)	0.44977 (10)	0.0228 (3)	
C4	0.42314 (12)	0.7347 (2)	0.49182 (11)	0.0241 (3)	
C5	0.44840 (12)	0.6872 (2)	0.58702 (11)	0.0242 (3)	
C6	0.55757 (11)	0.69292 (19)	0.64210 (9)	0.0204 (3)	
C7	0.86202 (10)	0.35212 (19)	0.54498 (9)	0.0182 (3)	
C8	0.87129 (10)	0.31449 (19)	0.44957 (9)	0.0175 (3)	
C9	0.88699 (11)	0.12938 (19)	0.42327 (10)	0.0199 (3)	
C10	0.89662 (11)	0.08955 (19)	0.33332 (10)	0.0220 (3)	
C11	0.88899 (13)	0.2343 (2)	0.26805 (10)	0.0248 (3)	
C12	0.87375 (12)	0.4168 (2)	0.29252 (10)	0.0248 (3)	
C13	0.86505 (11)	0.45797 (19)	0.38301 (9)	0.0203 (3)	
H3O	0.8478 (19)	0.650 (3)	0.4615 (8)	0.052 (6)*	
H4O	0.8920 (18)	−0.168 (3)	0.3363 (15)	0.050 (6)*	
H1N	0.8083 (15)	0.470 (2)	0.6862 (13)	0.034 (5)*	
H2	0.6718 (11)	0.834 (2)	0.4758 (12)	0.026 (4)*	
H3	0.4885 (15)	0.824 (3)	0.3850 (7)	0.031 (5)*	
H4	0.3471 (18)	0.729 (3)	0.4543 (14)	0.033 (5)*	
H5	0.3922 (12)	0.651 (3)	0.6166 (12)	0.032 (5)*	
H6	0.5728 (13)	0.657 (2)	0.7068 (7)	0.024 (4)*	
H7	0.8721 (15)	0.2494 (17)	0.5872 (10)	0.019 (4)*	
H9	0.8924 (15)	0.031 (2)	0.4681 (10)	0.028 (4)*	
H11	0.8879 (18)	0.201 (3)	0.2057 (8)	0.045 (6)*	
H12	0.8656 (15)	0.515 (2)	0.2476 (11)	0.032 (5)*	

### Atomic displacement parameters (Å^2^)

**Table:**

	*U*^11^	*U*^22^	*U*^33^	*U*^12^	*U*^13^	*U*^23^
S1	0.01696 (19)	0.01935 (18)	0.01461 (19)	−0.00098 (10)	0.00306 (13)	−0.00294 (10)
N1	0.0212 (5)	0.0213 (5)	0.0157 (5)	0.0023 (4)	0.0070 (4)	0.0008 (4)
N2	0.0174 (5)	0.0235 (6)	0.0161 (5)	0.0000 (4)	0.0066 (4)	−0.0005 (4)
O1	0.0261 (5)	0.0312 (6)	0.0144 (5)	0.0025 (4)	0.0025 (4)	−0.0053 (4)
O2	0.0229 (5)	0.0226 (5)	0.0288 (5)	−0.0048 (4)	0.0065 (4)	−0.0010 (4)
O3	0.0434 (6)	0.0195 (5)	0.0232 (5)	0.0037 (4)	0.0147 (5)	0.0033 (4)
O4	0.0461 (7)	0.0225 (5)	0.0318 (6)	−0.0037 (4)	0.0249 (5)	−0.0047 (4)
C1	0.0166 (6)	0.0173 (6)	0.0150 (6)	0.0003 (4)	0.0037 (5)	−0.0022 (4)
C2	0.0210 (6)	0.0231 (6)	0.0164 (6)	0.0009 (5)	0.0069 (5)	−0.0010 (5)
C3	0.0235 (7)	0.0274 (7)	0.0163 (6)	0.0028 (5)	0.0033 (5)	−0.0013 (5)
C4	0.0187 (7)	0.0266 (7)	0.0247 (7)	−0.0007 (5)	0.0020 (6)	−0.0034 (5)
C5	0.0208 (6)	0.0255 (7)	0.0281 (7)	−0.0030 (5)	0.0097 (5)	−0.0006 (6)
C6	0.0225 (6)	0.0219 (6)	0.0176 (6)	−0.0010 (5)	0.0069 (5)	0.0010 (5)
C7	0.0162 (6)	0.0214 (6)	0.0175 (6)	0.0003 (5)	0.0054 (5)	0.0018 (5)
C8	0.0148 (6)	0.0219 (6)	0.0167 (6)	0.0003 (5)	0.0058 (4)	0.0011 (5)
C9	0.0200 (6)	0.0207 (6)	0.0211 (6)	0.0002 (5)	0.0091 (5)	0.0018 (5)
C10	0.0220 (6)	0.0220 (6)	0.0253 (7)	−0.0025 (5)	0.0118 (5)	−0.0027 (5)
C11	0.0286 (7)	0.0300 (7)	0.0187 (7)	−0.0033 (5)	0.0116 (6)	−0.0017 (5)
C12	0.0298 (7)	0.0260 (7)	0.0201 (7)	−0.0009 (6)	0.0095 (5)	0.0051 (5)
C13	0.0202 (6)	0.0200 (6)	0.0214 (7)	0.0011 (5)	0.0071 (5)	0.0010 (5)

### Geometric parameters (Å, °)

**Table:**

S1—O2	1.4304 (10)	C4—C5	1.389 (2)
S1—O1	1.4415 (10)	C4—H4	0.97 (2)
S1—N1	1.6567 (12)	C5—C6	1.395 (2)
S1—C1	1.7602 (13)	C5—H5	0.959 (9)
N1—N2	1.3920 (15)	C6—H6	0.953 (9)
N1—H1N	0.85 (1)	C7—C8	1.4607 (17)
N2—C7	1.2874 (17)	C7—H7	0.948 (9)
O3—C13	1.3733 (16)	C8—C13	1.4051 (18)
O3—H3O	0.85 (1)	C8—C9	1.4090 (18)
O4—C10	1.3677 (16)	C9—C10	1.3870 (19)
O4—H4O	0.85 (1)	C9—H9	0.953 (9)
C1—C2	1.3963 (18)	C10—C11	1.397 (2)
C1—C6	1.3976 (18)	C11—C12	1.382 (2)
C2—C3	1.3904 (19)	C11—H11	0.943 (9)
C2—H2	0.958 (9)	C12—C13	1.3928 (19)
C3—C4	1.392 (2)	C12—H12	0.950 (9)
C3—H3	0.951 (9)		
			
O2—S1—O1	120.59 (6)	C6—C5—H5	118.4 (11)
O2—S1—N1	107.38 (6)	C5—C6—C1	118.40 (12)
O1—S1—N1	103.14 (6)	C5—C6—H6	118.2 (10)
O2—S1—C1	108.44 (6)	C1—C6—H6	123.4 (10)
O1—S1—C1	108.51 (6)	N2—C7—C8	118.90 (12)
N1—S1—C1	108.14 (6)	N2—C7—H7	124.2 (10)
N2—N1—S1	112.33 (9)	C8—C7—H7	116.9 (10)
N2—N1—H1N	118.3 (13)	C13—C8—C9	118.90 (12)
S1—N1—H1N	110.4 (13)	C13—C8—C7	121.83 (12)
C7—N2—N1	118.47 (11)	C9—C8—C7	119.28 (12)
C13—O3—H3O	110.2 (17)	C10—C9—C8	120.55 (12)
C10—O4—H4O	110.5 (16)	C10—C9—H9	119.8 (11)
C2—C1—C6	121.94 (12)	C8—C9—H9	119.6 (11)
C2—C1—S1	118.65 (10)	O4—C10—C9	123.53 (13)
C6—C1—S1	119.40 (10)	O4—C10—C11	116.96 (12)
C3—C2—C1	118.80 (12)	C9—C10—C11	119.50 (13)
C3—C2—H2	120.7 (11)	C12—C11—C10	120.81 (13)
C1—C2—H2	120.5 (11)	C12—C11—H11	121.8 (14)
C2—C3—C4	119.77 (13)	C10—C11—H11	117.1 (14)
C2—C3—H3	118.6 (11)	C11—C12—C13	119.98 (13)
C4—C3—H3	121.6 (11)	C11—C12—H12	121.0 (12)
C5—C4—C3	121.10 (13)	C13—C12—H12	118.9 (12)
C5—C4—H4	119.2 (12)	O3—C13—C12	118.16 (12)
C3—C4—H4	119.7 (12)	O3—C13—C8	121.59 (12)
C4—C5—C6	119.98 (13)	C12—C13—C8	120.25 (12)
C4—C5—H5	121.6 (11)		
			
O2—S1—N1—N2	−50.34 (10)	S1—C1—C6—C5	179.56 (10)
O1—S1—N1—N2	−178.71 (8)	N1—N2—C7—C8	−179.90 (10)
C1—S1—N1—N2	66.48 (10)	N2—C7—C8—C13	2.14 (19)
S1—N1—N2—C7	−163.01 (10)	N2—C7—C8—C9	−177.89 (12)
O2—S1—C1—C2	27.78 (12)	C13—C8—C9—C10	0.28 (19)
O1—S1—C1—C2	160.41 (10)	C7—C8—C9—C10	−179.68 (12)
N1—S1—C1—C2	−88.36 (11)	C8—C9—C10—O4	178.20 (12)
O2—S1—C1—C6	−152.52 (10)	C8—C9—C10—C11	−1.0 (2)
O1—S1—C1—C6	−19.89 (12)	O4—C10—C11—C12	−178.16 (14)
N1—S1—C1—C6	91.34 (11)	C9—C10—C11—C12	1.0 (2)
C6—C1—C2—C3	−0.1 (2)	C10—C11—C12—C13	−0.5 (2)
S1—C1—C2—C3	179.64 (10)	C11—C12—C13—O3	179.51 (13)
C1—C2—C3—C4	0.8 (2)	C11—C12—C13—C8	−0.2 (2)
C2—C3—C4—C5	−0.7 (2)	C9—C8—C13—O3	−179.42 (12)
C3—C4—C5—C6	−0.1 (2)	C7—C8—C13—O3	0.5 (2)
C4—C5—C6—C1	0.8 (2)	C9—C8—C13—C12	0.32 (19)
C2—C1—C6—C5	−0.8 (2)	C7—C8—C13—C12	−179.71 (12)

### Hydrogen-bond geometry (Å, °)

**Table:**

*D*—H···*A*	*D*—H	H···*A*	*D*···*A*	*D*—H···*A*
O3—H3O···N2	0.85 (1)	1.82 (2)	2.562 (2)	145 (2)
O4—H4O···O3^i^	0.85 (1)	1.85 (1)	2.698 (2)	175 (2)
N1—H1N···O1^ii^	0.85 (1)	2.05 (1)	2.897 (2)	171 (2)

Symmetry codes: (i) *x*, *y*−1, *z*; (ii) −*x*+3/2, *y*−1/2, −*z*+3/2.
